# Supplementary material for: The Israeli acute paralysis virus IRES captures host ribosomes by mimicking a ribosomal state with hybrid tRNAs
Source: EMBO J. 2019 Oct 14;38(21):e102226. doi: 10.15252/embj.2019102226 (PMC6826211; doi:10.15252/embj.2019102226)
Supplement: Supplementary file 1 — Appendix [file EMBJ-38-e102226-s001.pdf]

Appendix for

The Israeli Acute Paralysis Virus IRES captures host ribosomes by mimicking a ribosomal state with hybrid tRNAs.

Francisco Acosta-Reyes, Ritam Neupane, Joachim Frank* and Israel S. Fernández*

* Corresponding author email:

jf2192@cumc.columbia.edu (JF) and isf2106@cumc.columbia.edu (ISF)

This file contains:

Appendix Figures S1-S7

Appendix Table S1

Appendix Figures S1-S7

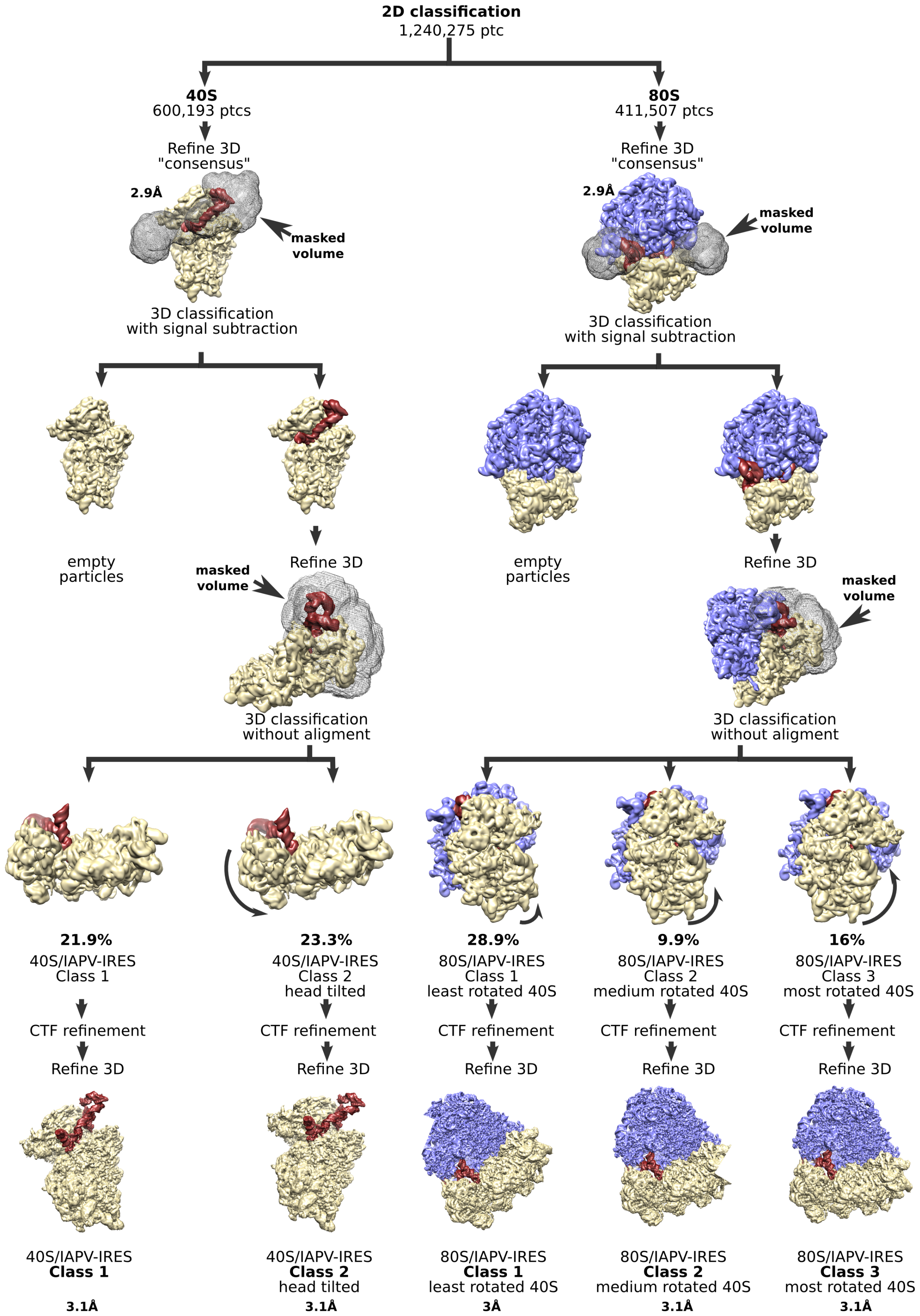

Figure S1. Classification scheme followed for the pre-translocation dataset.

(Top), 40S and 80S particles were separated by reference-free 2D classification. Homogeneous subgroups of 40S and 80S particles were subjected to two steps of masked classification intercalated with refinements to, on a first instance, identified those particles with IAPV-IRES and, in a second instance, distinguish among the groups with IAPV-IRES, different conformations. (Bottom), New features implemented in Relion3.0 (Zivanov, Nakane et al., 2018) such as contrast transfer values refinement allowed extending the resolution to close to 3Å for the five populations.

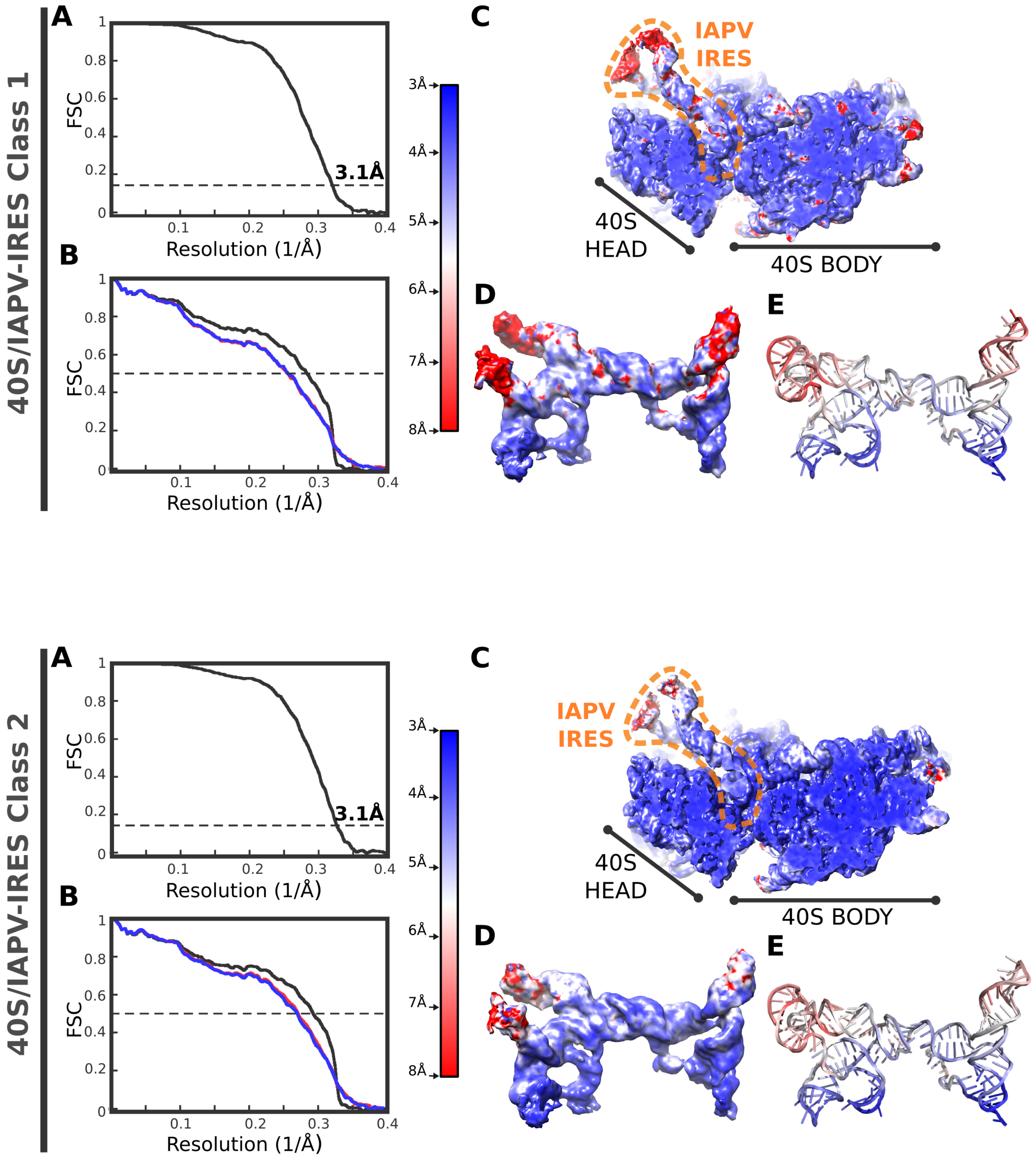

40S/IAPV-IRES Class 1
A
FSC
Resolution (1/Å)
3.1Å
B
FSC
Resolution (1/Å)
3Å
4Å
5Å
6Å
7Å
8Å
C
IAPV IRES
40S HEAD
40S BODY
D
E
40S/IAPV-IRES Class 2
A
FSC
Resolution (1/Å)
3.1Å
B
FSC
Resolution (1/Å)
3Å
4Å
5Å
6Å
7Å
8Å
C
IAPV IRES
40S HEAD
40S BODY
D
E

Figure S2: Fourier Shell Correlation curves and local resolution estimation for the 40S/IAPV-IRES complex classes.
For each class:

(A) Fourier Shell Correlation (FSC) computed for the two half maps of the final subset of particles after classification for the first class identified for the 40S/IAPV-IRES complex. The resolution is estimated to be 3.1Å using the 0.143 criterion (Rosenthal & Henderson, 2003). (B) Map-versus-model cross validation FSC. The final model was validated using standard procedures: FSC of the refined model against half map 1 (blue) overlaps with the FSC against half map 2 (red, not included in the refinement). The black curve corresponds to the FSC of the final model against the final map. (C) Slice through the final, unsharpened map colored according to the local resolution as reported by RESMAP (Kucukelbir, Sigworth et al., 2014). (D) Close-up views of the final density colored according to local resolution values as in (C) for the IAPV-IRES. (E) Final refined IAPV-IRES model colored according to the estimated B-factors in $Å^2$ computed by REFMAC (Murshudov, Vagin et al., 1997). Specific values for estimated resolutions are indicated for each class.

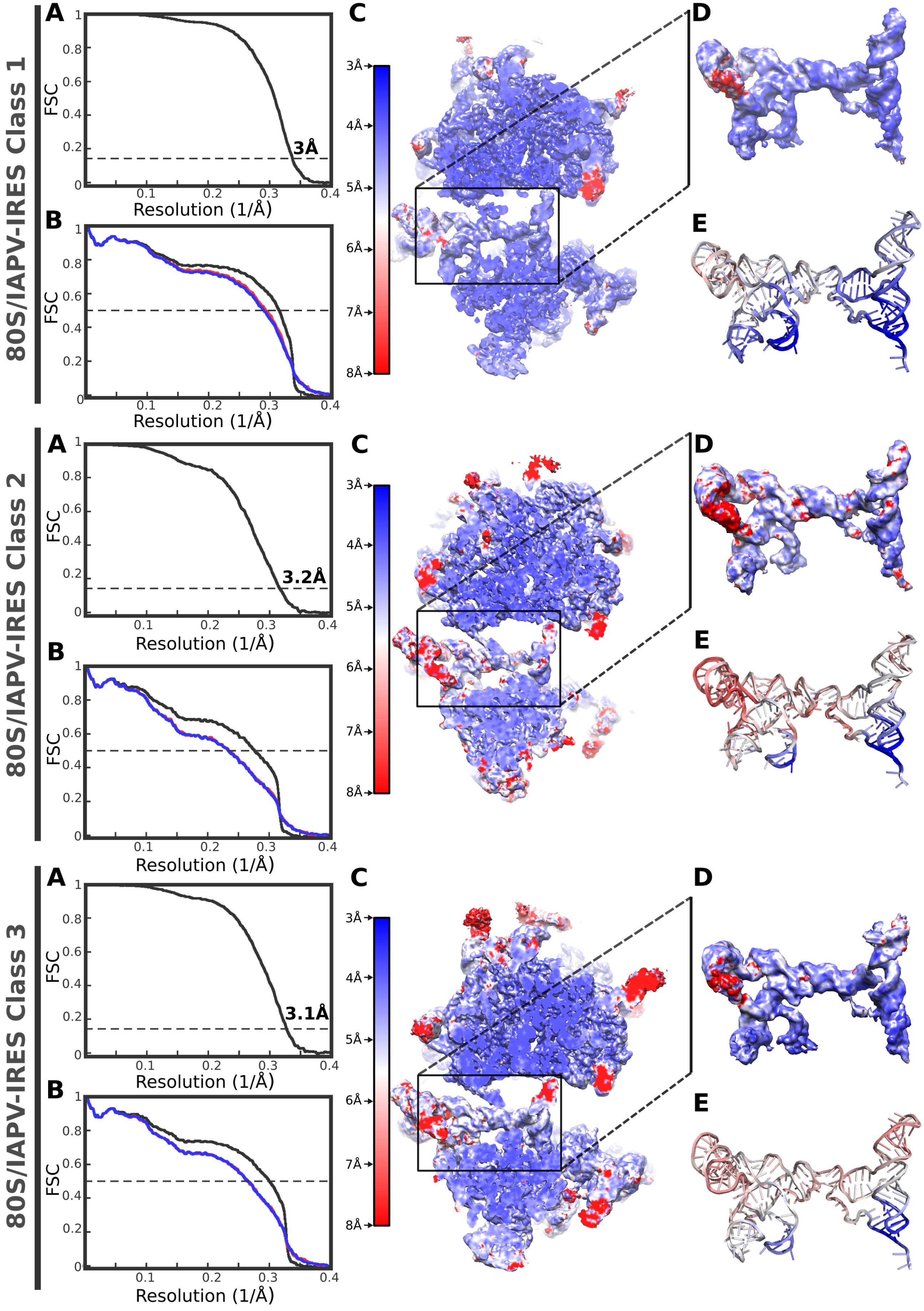

80S/IAPV-IRES Class 1
A
B
C
D
E
FSC
Resolution (1/Å)
3Å
3Å
4Å
5Å
6Å
7Å
8Å
80S/IAPV-IRES Class 2
3.2Å
80S/IAPV-IRES Class 3
3.1Å

Figure S3: Fourier Shell Correlation curves and local resolution estimation for the 80S/IAPV-IRES complex classes.
For each class:

(A) Fourier Shell Correlation (FSC) computed for the two half maps of the final subset of particles after classification for the first class identified for the 80S/IAPV-IRES complex. The resolution are estimated to be 3.0-3.2Å using the 0.143 criterion (Rosenthal & Henderson, 2003). (B) Map-versus-model cross validation FSC. The final model was validated using standard procedures: FSC of the refined model against half map 1 (blue) overlaps with the FSC against half map 2 (red, not included in the refinement). The black curve corresponds to the FSC of the final model against the final map. (C) Slice through the final, unsharpened map colored according to the local resolution as reported by RESMAP (Kucukelbir et al., 2014). (D) Close-up views of the final density colored according to local resolution values as in (C) for the IAPV-IRES. (E) Final refined IAPV-IRES model colored according to the estimated B-factors in Å^2 computed by REFMAC. Specific values for estimated resolutions are indicated for each class.

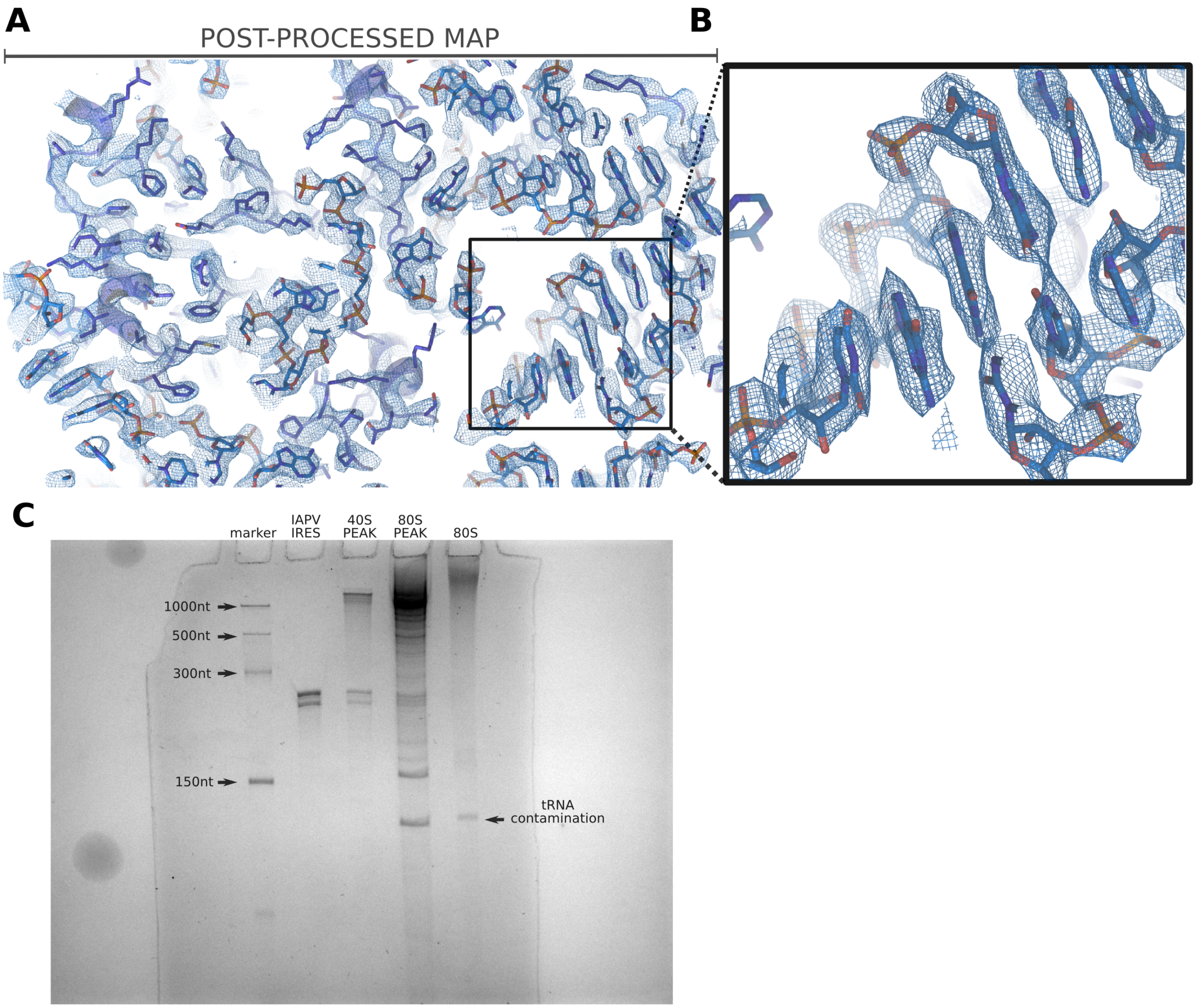

Figure S4: Post-processed map and UREA-PAGE.

(A) and (B) Slice through the 80S/IAPV-IRES class 1 post-processed map centered around the 60S. Map features expected for a 3Å map like protein side chains and base separation for nucleic acids can be appreciated. (C). UREA-PAGE analysis of 40S and 80S peaks resolved in a overnight sucrose gradient run. Bands corresponding to the purified IAPV-IRES RNA can be appreciated in both 40S and 80S peaks.

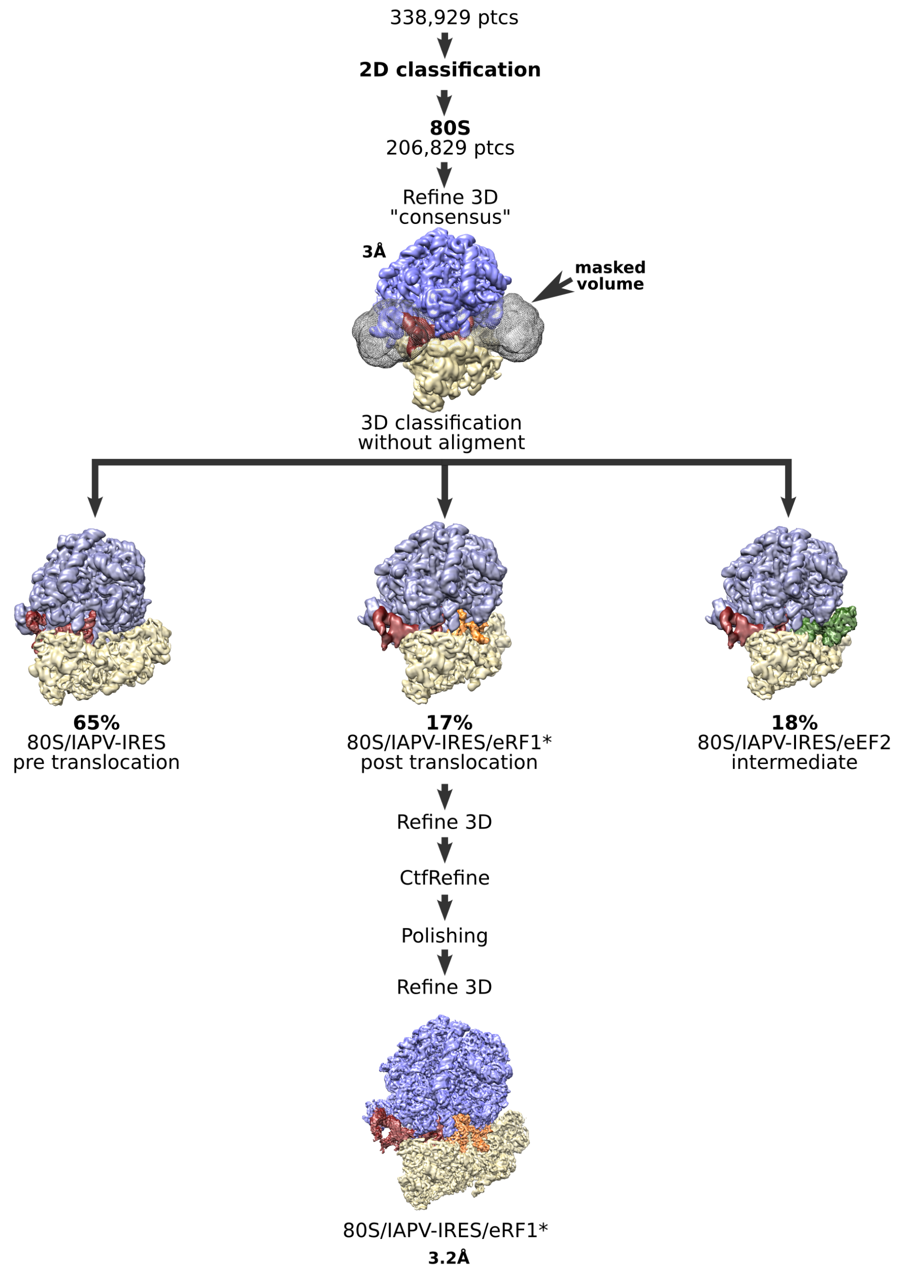

Figure S5: Classification scheme followed for the post-translocation dataset.

(Top), after 2D and 3D masked classifications, three populations of particles corresponding to pre-translocation, post-translocation and an intermediate state with eEF2 could be identified in the dataset. (Bottom), Contrast transfer values refinement and Bayesian particle polishing implemented in Relion3.0 (Zivanov et al., 2018) allowed the extension of the resolution for the post-translocation state to 3.2Å.

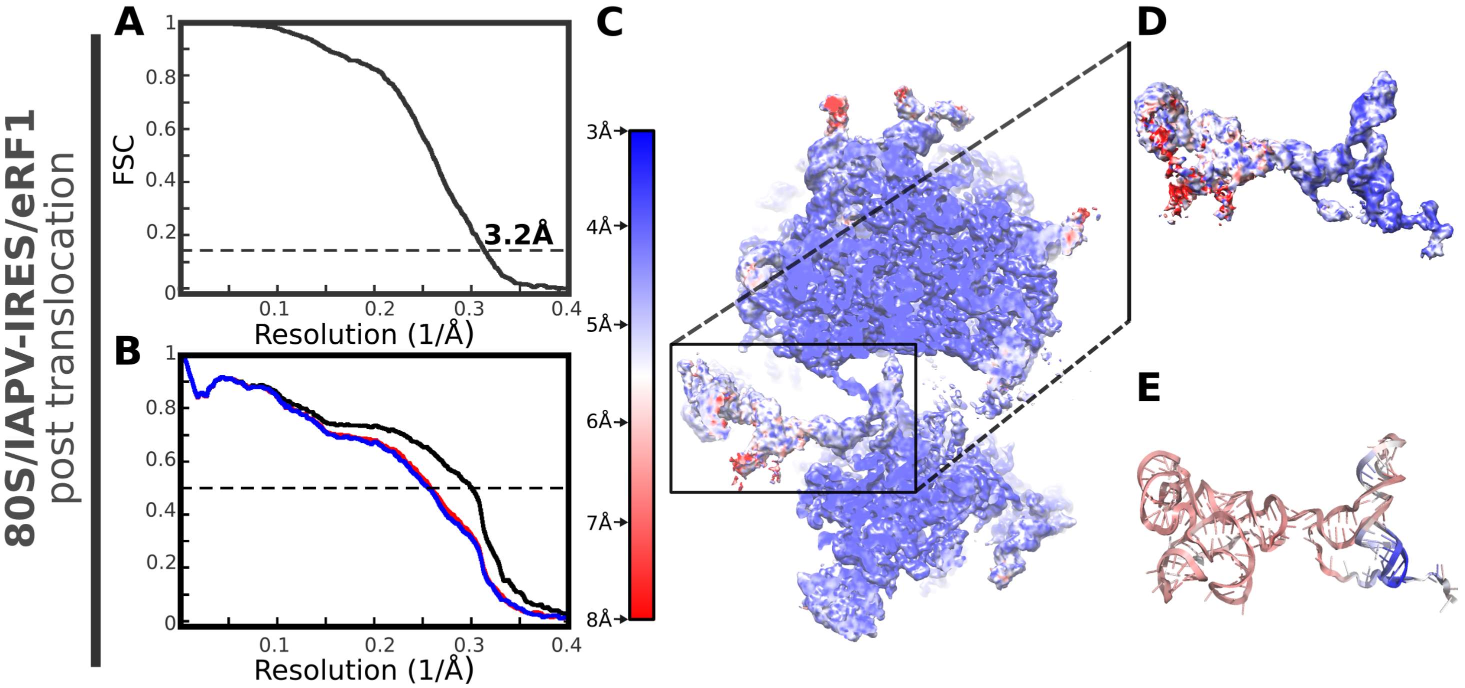

Figure S6: Fourier Shell Correlation curves and local resolution estimation for the post-translocated 80S/IAPV-IRES/eRF1* complex.

(A) Fourier Shell Correlation (FSC) computed for the two half maps of the final subset of particles after classification for the first class identified for the 80S/IAPV-IRES complex. The resolution is estimated to be 3.2Å using the 0.143 criterion (Rosenthal & Henderson, 2003). (B) Map-versus-model cross validation FSC. The final model was validated using standard procedures: FSC of the refined model against half map 1 (blue) overlaps with the FSC against half map 2 (red, not included in the refinement). The black curve corresponds to the FSC of the final model against the final map. (C) Slice through the final, unsharpened map colored according to the local resolution as reported by RESMAP (Kucukelbir et al., 2014). (D) Close-up views of the final density colored according to local resolution values as in (C) for the IAPV-IRES. (E) Final refined IAPV-IRES model colored according to the estimated B-factors in Å^2 computed by REFMAC (Murshudov et al., 1997).

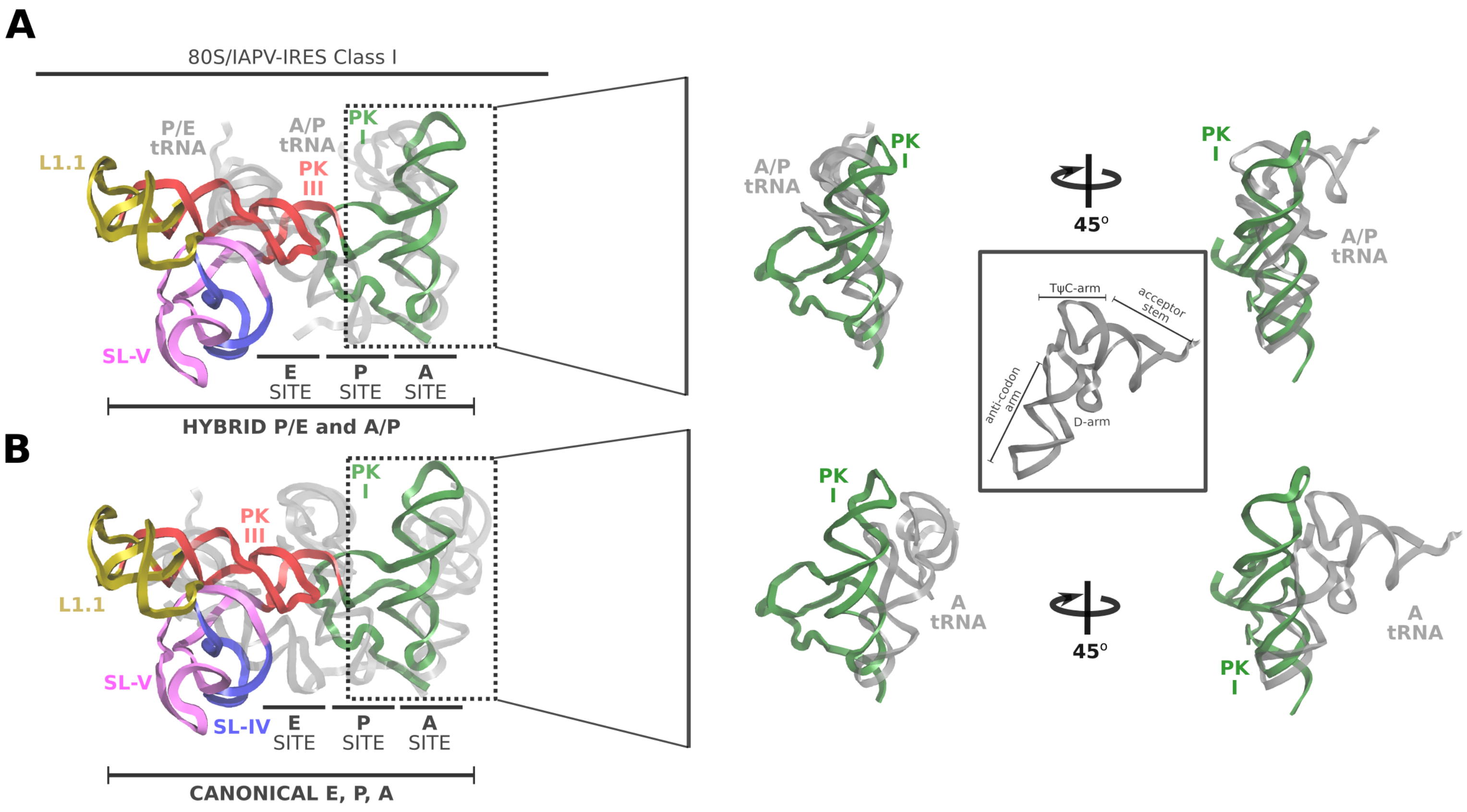

Figure S7: tRNA mimicry in the 80S/IAPV-IRES pre-translocation state.

(A) and (B) Superposition of IAPV-IRES in the pre-translocation state class 1 with tRNAs in hybrid-states (A) and with canonical tRNAs (B). On the right, close-up view of the superposition for the PKI domain of the IAPV- IRES in two orientations. It can be appreciated the co-axial unit formed by SL-III and the tRNA/mRNA-like domain of the IRES occupies a space similar to a hybrid A/P tRNA. The tRNA mimicry is however not complete as the acceptor stem of the tRNA is not accounted by any element of the IRES.

Data collection	Collection Polara-F30					Collection Titan-Krios
Micrographs	9973					4887
Picked Particles	1240275					338929
Class 2D particles	1011700					206829
Voltage (KV)	300					300
Defocus range* (µm)	0.4-3.5					1.2-4.9
Defocus mean (µm)	1.5					2.2
Pixel size (Å/pixel)	1.233					1.0605
Frames / Movie	40					40
Electron dose (e-/Å^2)	42.1					56.9
Electron dose per frame (e-/Å^2)	1.0525					1.4225
**Stucture**						
Component	40S/IAPV-IRES		80S/IAPV-IRES pre-translocated			80S/IAPV-IRES post-translocated
	**Class-1**	**Class-2**	**Class-1**	**Class-2**	**Class-3**	**Class-Post T.**
Particles	91056	96826	120176	40701	68697	27658
FSC 0.143 (Å)	3.1	3.1	3	3.1	3.1	3.2
Map sharpening (Å^2)	-70.3	-66.6	-67.5	-54.1	-65.1	-75.4
**Refinement**						
Program/Protocol	Refmac5 / Phenix	Refmac5 / Phenix	Refmac5 / Phenix	Refmac5 / Phenix	Refmac5 / Phenix	Refmac5 / Phenix
Used in refinement (Å)	3.1	3.1	3.1	3.1	3.1	3.2
Average B-factors (Å^2)	128.29	125.96	77.57	89.34	63.32	123.21
Avg B-fac Prot/RNA (Å^2)	129.77/126.89	127.35/124.62	83.85/72.86	93.28/86.39	65.82/61.44	126.50/120.66
R.m.s deviations:						
Bonds (Å)	0.0037	0.0031	0.0031	0.0027	0.0027	0.0032
Angles (deg)	1.1622	1.1208	1.1038	1.0408	1.0511	1.083
**Validation**						
Molprobity score	1.86	1.65	1.75	1.68	1.74	1.63
Clashcore, all atoms	2.09	2.29	2.23	2.07	2.25	2.11
Rotamer outliers (%)	2.77	1.77	2.16	1.6	2.04	1.5
Ramachandran plot:						
Outliers (%)	0.86	0.67	0.63	0.52	0.62	0.61
Favored (%)	90.36	92.75	91.79	90.01	91.43	91.32
**Composition**						
Non hydrogen atoms	79372	79379	215972	215966	215976	219296
Protein residues	4862	4862	11491	11490	11490	11892
RNA bases	1900	1900	5766	5766	5766	5768
Ligands	0	0	0	0	0	0
**Accession codes**						
EMDB	20248	20249	20255	20256	20257	20258
PDB	6P4G	6P4H	6P5I	6P5J	6P5K	6P5N

Table S1:

Data collection, model refinement and validation statistics. *Defocus range reported by GCT

Supplemental References:

Kucukelbir A, Sigworth FJ, Tagare HD (2014) Quantifying the local resolution of cryo-EM density maps. *Nat Methods* 11: 63-5

Murshudov GN, Vagin AA, Dodson EJ (1997) Refinement of macromolecular structures by the maximum-likelihood method. *Acta Crystallogr D Biol Crystallogr* 53: 240-55

Rosenthal PB, Henderson R (2003) Optimal determination of particle orientation, absolute hand, and contrast loss in single-particle electron cryomicroscopy. *J Mol Biol* 333: 721-45

Zivanov J, Nakane T, Forsberg BO, Kimanius D, Hagen WJ, Lindahl E, Scheres SH (2018) New tools for automated high-resolution cryo-EM structure determination in RELION-3. *Elife* 7
