# Supplementary material for: The Israeli acute paralysis virus IRES captures host ribosomes by mimicking a ribosomal state with hybrid tRNAs
Source: EMBO J. 2019 Oct 14;38(21):e102226. doi: 10.15252/embj.2019102226 (PMC6826211; doi:10.15252/embj.2019102226)
Supplement: Supplementary file 2 — Movie EV1 [file EMBJ-38-e102226-s002.zip › Figure_Legend_for_Movie_EV1.docx]

Figure Legend for Movie EV1:

**Conformational transitions of IAPV-IRES along its**

**movement through the ribosome.**

IAPV-IRES binds initially to the ribosome inserting the PKI (green) in the

A site mimicking a hybrid A/P-tRNA. Once translocated, PKI (green) is

placed in the ribosomal P site with a configuration reminiscent of a canoni-

cal P tRNA. Along this movement, needed to place the first coding codon of

the viral messenger (black) in the A site, flexible regions of the IAPV-IRES

experiment conformational transitions in a context specific manner. IAPV-

IRES is colored according to the secondary structure motives indicated in

figure 1A and canonical tRNAs from PDBID 4V5D [5] are represented as

semi-transparent grey cartoons. Molecular transitions have been approxi-

mated by a linear morph using Chimera [6].
